# The Performance of Four Different Mineral Liners on the Transportation of Chlorinated Phenolic Compounds to Groundwater in Landfills

**DOI:** 10.1155/2015/171284

**Published:** 2015-11-29

**Authors:** Elanur Adar, Mehmet Sinan Bilgili

**Affiliations:** Department of Environmental Engineering, Faculty of Civil Engineering, Yildiz Technical University, Davutpasa, Esenler, 34220 Istanbul, Turkey

## Abstract

The aim of this study was to investigate the efficiency of four different mineral liners (clay, bentonite, kaoline, and zeolite) which could be utilized to prevent the transport of phenolic compounds to groundwater through alternative liner systems. Four laboratory-scale HDPE reactors with 80 cm height and 40 cm inner diameter were operated for a period of 180 days. Results indicated that the transport of mono- or dichlorophenols is significantly prevented by the liner systems used, while the transport of highly chlorinated phenolic compounds cannot be prevented by the landfill liner system effectively. Highly chlorinated phenolic compounds in groundwater can be found in higher concentrations than the leachate, as a result of the degradation and transformation of these compounds. Thus, the analysis of highly chlorinated phenolic compounds such as 2,4,6-TCP, 2,3,6-TCP, 3,4,5-TCP, and PCP is of great significance for the studies to be conducted on the contamination of groundwater around landfills.

## 1. Introduction

In recent years, the characteristics of wastes in MSW landfills have varied according to the changing habits of consumers. The characteristics of leachate generated from sanitary landfill sites vary according to the waste compounds disposed and the physical, chemical, and biological processes occurring in landfills. Household hazardous wastes such as batteries, paints, oils, electrical products, and pharmaceuticals have a negative impact on environmental and human health [1]. The presence of organic pollutants such as halogenated aliphatic compounds, aromatic hydrocarbons, phenols, and pesticides in leachate indicates that hazardous wastes are also disposed in landfills [2, 3].

In studies conducted to determine the pollutants in both leachate and the leachate contaminated groundwater, numerous hazardous substances were identified [4–7]. Phenolic compounds are classified as hazardous substances due to their high toxicity, corrosiveness, flammability, reactivity, and carcinogenic, mutagenic, and ecotoxic properties. Because of these characteristics, phenolic compounds are named as priority pollutants by the US EPA [8]. Since phenolic compounds create unpleasant taste and odor in water even at very low concentrations, the prevention of their migration to groundwater is of great significance. Landfill leachate is one of the major sources of groundwater contamination. Thus, in modern sanitary landfill sites, liner systems are used to prevent the migration of leachate contaminants to groundwater. Liner systems generally consist of a compacted clay liner (0.3–1.5 m) and a 1–2.5 mm HDPE (high density polyethylene) geomembrane [9]. The pollutant transfer from landfill leachate to groundwater is generally realized in two ways: geomembrane defects and diffusion [10–13].

Diffusion constitutes the main transport mechanism for the pollutant transport through landfill liners to groundwater [14]. While inorganic pollutants are generally transported through advection or diffusion or by means of their union through geomembrane and liner defects, the transport of volatile organic compounds (VOCs) is realized through diffusion [11, 15–18]. The fate and transport of organic pollutants are affected by processes such as advective, dispersive, and diffusive mass transport through the mineral layer, chemical reactions within the soil solution (evaporation, hydrolysis), interaction between the soil solution and the soil pollutants (e.g., adsorption, ion exchange, and precipitation), and biodegradation [19, 20]. These processes are complex and related with the structure of the waste as well as the material used and the design of the liner system [20].

In recent years, there has been an increase in the number of studies conducted on the effects of leachate on the contamination of water resources [2, 3, 21, 22]. The studies have investigated the transport of conventional parameters such as chloride, ammonia, and heavy metals [23–25] and some volatile compounds [9, 13, 26–29] through landfill liners. However, there are only few studies on the transport of phenols and chlorophenols through landfill liners [30, 31]. Phenolic compounds in leachate are generated as a result of the degradation of the phenol-containing compounds in municipal solid waste [27, 32, 33]. Of the four isomers of trichlorophenols investigated in this study (2,4,6-TCP, 2,4,5-TCP, 2,3,6-TCP, and 3,4,5-TCP), the 2,4,5- and 2,4,6-isomers are listed on the US EPA's priority pollutants list. The latter has been included in Directive 76/464/EEC (European Economic Community) as a dangerous substance discharged into the aquatic environment [34, 35].

The aim of this study was to investigate the performance of different liner materials for the prevention of transport of phenolic compounds to groundwater. Four different liners (clay, bentonite, zeolite, and kaoline) with the same thickness (20 cm) were used in the simulated reactors. Phenol, 2,4-dichlorophenol (2,4-DCP), 2,6-dichlorophenol (2,6-DCP), 2,4,6-trichlorophenol (2,4,6-TCP), 2,4,5-trichlorophenol (2,4,5-TCP), 2,3,6-trichlorophenol (2,3,6-TCP), 3,4,5-trichlorophenol (3,4,5-TCP), and pentachlorophenol (PCP) concentrations in leachate and groundwater samples were identified and the performance of different liner materials was determined in order to evaluate the transport of phenolic compounds.

## 2. Material and Methods

### 2.1. Reactors and Liner Systems

**Figure 1:**
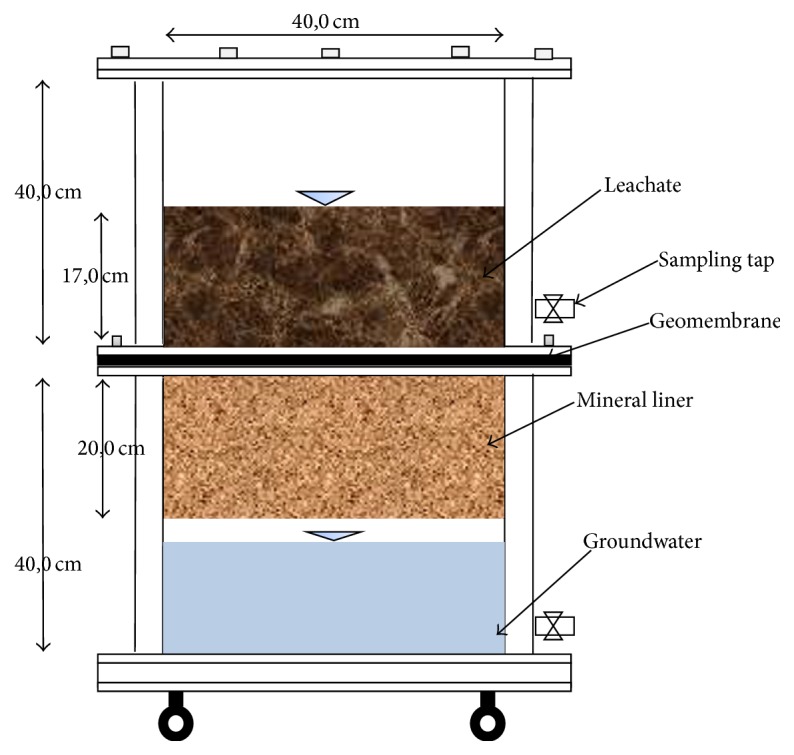
Reactors used in the study.

Four lab-scale reactors with a height of 80 cm and an inner diameter of 40 cm were used in order to determine the transport of phenolic compounds in landfill leachate through liner systems (Figure 1). The study was conducted at 25°C. During the study, air was not fed into reactors. The HDPE reactors were comprised of two parts, each with a height of 40 cm.

The upper part of the liner was filled with 20 liters of leachate (app. 17 cm height) obtained from Odayeri Sanitary Landfill located on the European Side of Istanbul, Turkey. According to the US and the European standards, the maximum leachate load on landfill liner should be 20–50 cm [36] to improve the performance of the composite liner and to ensure the efficient pump operation. However there is no limit for leachate height in landfill liners in the Landfill Directive used in Turkey. According to this regulation, the hydraulic conductivity of the liner system must be 10^−9^ m/s in landfills. The general waste composition and physicochemical characteristics of solid wastes generated in Istanbul were given in our previous study [37].

The liner system of reactors is comprised of 2 mm thick HDPE geomembrane and 20 cm thick natural minerals (R1: clay, R2: bentonite, R3: kaoline, and R4: zeolite). The liner systems are compacted to prevent the possibilities of leakage at commissioning stage. Mineral layers are filled by the optimum water content obtained from soil analysis. The lower part of the reactors was filled with 25 liters of distilled water representing groundwater.

### 2.2. Analysis

Leachate characterization was realized before the upper parts of the reactors were filled. pH, electrical conductivity, COD, BOD, TOC, CI^−^, TKN, NH_3_-N, and SO_4_^−2^ analysis were conducted according to the standard methods of APHA (2005) [38]. Groundwater samples were collected from reactors to be analyzed biweekly for the first two months and monthly in the following period. Additionally, leachate samples were collected bimonthly for the analysis of the phenolic compounds in order to determine the change in the concentrations as a result of biological activities.

To determine phenol and chlorinated phenolic compounds in leachate and groundwater samples, solid phase microextraction (SPME) method was used as conducted by Ribeiro et al. [39]. The extraction and analysis conditions are given in our previous study [34]. Phenol and chlorophenol concentrations were determined with a Varian 3900 GC-FID gas chromatograph. All of the experiments were repeated 2 times for all parameters and the results are given as an average of these two measurements.

pH, hydraulic conductivity, soil classification, compaction test [40], cation exchange capacity [41], and TOC-TN analyses were performed for the characterization of the mineral materials. TOC-TN analysis was conducted by using Hach Lange IL 550 TOC-TN model apparatus following the thermal oxidation method at high temperature.

## 3. Results and Discussions

### 3.1. Leachate Characterization

The mean values of leachate characterization analyses realized on 3 different leachate samples are given in Table 1. In general, the leachate samples demonstrated the characteristics of a middle-aged landfill leachate [42]. Phenol and 2,4-DCP concentrations were higher in leachate samples. The high phenol concentrations can be explained by the reduction of all phenolic compounds to phenol under anaerobic conditions and the slow degradation of phenol in anaerobic landfills.

In the result of repeated experiments, as the values for conventional parameters were near to each other, determined values for phenolic compounds were not near due to volatile properties of phenolic compounds. The studies conducted to determine the phenolic compounds in the leachate also demonstrated that different derivatives of monochlorophenol and pentachlorophenol were present. In the study conducted by Öman and Junestedt [42], the maximum phenol concentration was found to be 4.1 *μ*g/L while the maximum concentration of chlorophenol compounds was determined to 23 *μ*g/L. Jiménez et al. [43] reported that PCP, 2,4,6-TCP, 2,3,4,6-TeCP, 2,4-DCP, and 3,4-DCP concentrations in the leachate were 0.01–3000, 0.08–1.87, 0.08–20.4, 0.34–12.8, and 0.27–14.3 *μ*g/L, respectively. Another study conducted by Ozkaya [44] revealed that 2,4-DCP, 2,6-DCP, 2,3,4-TCP, 2,3,4,5-TeCP, and 2,3,4,6-TeCP were found in acidogenic leachate while only 2,4-DCP was found in methanogenic leachate.

### 3.2. Soil Characterization

The results of the characterization experiments of clay, bentonite, kaoline, and zeolite used as landfill liner materials in this study are given in Table 2. According to Akyildiz [45], the high pH levels of the four minerals used in the study are the characteristic of compacted soils. Soils are best compacted at their optimum water content, which results in the lowest hydraulic conductivity. According to the results of the compaction and the falling head permeability tests, bentonite was determined to be the mineral with the lowest hydraulic conductivity because of its high water-holding capacity. Furthermore, permeability increases as the size of the particles in the mineral layer increases depending on the silt content. The cation exchange capacity of bentonite is higher than that for the other minerals employed in the study. Accordingly, bentonite is observed to have a higher adsorption capacity.

### 3.3. Phenolic Compounds in Groundwater and Leachate Samples

**Figure 2:**
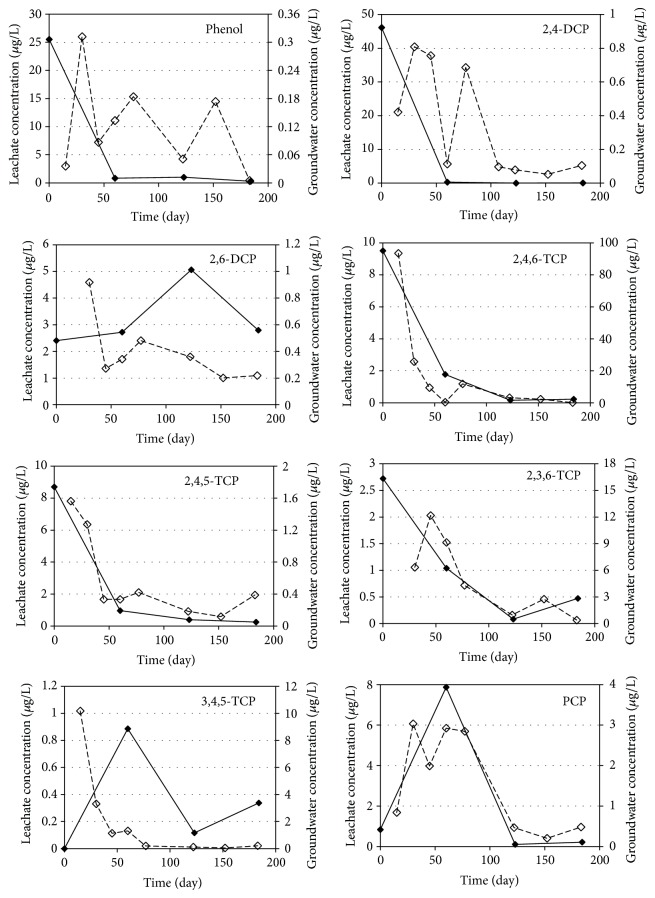
Variations of the phenolic compounds in groundwater and leachate samples of R1 reactor (——: leachate concentration, - - - - -: groundwater concentration).

**Figure 3 fig3:**
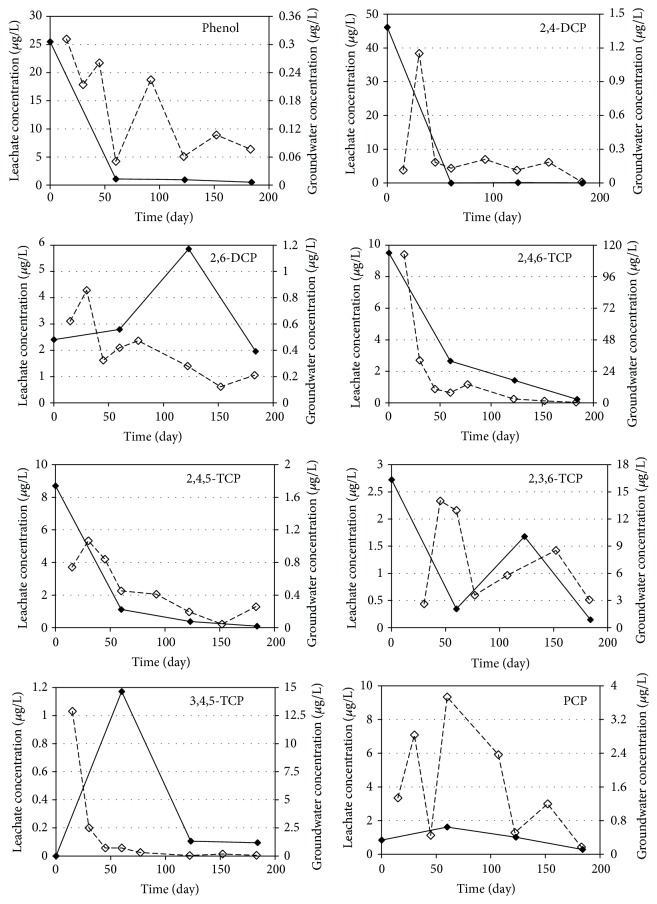
Variations of the phenolic compounds in groundwater and leachate samples of R2 reactor (——: leachate concentration, - - - - -: groundwater concentration).

**Figure 4 fig4:**
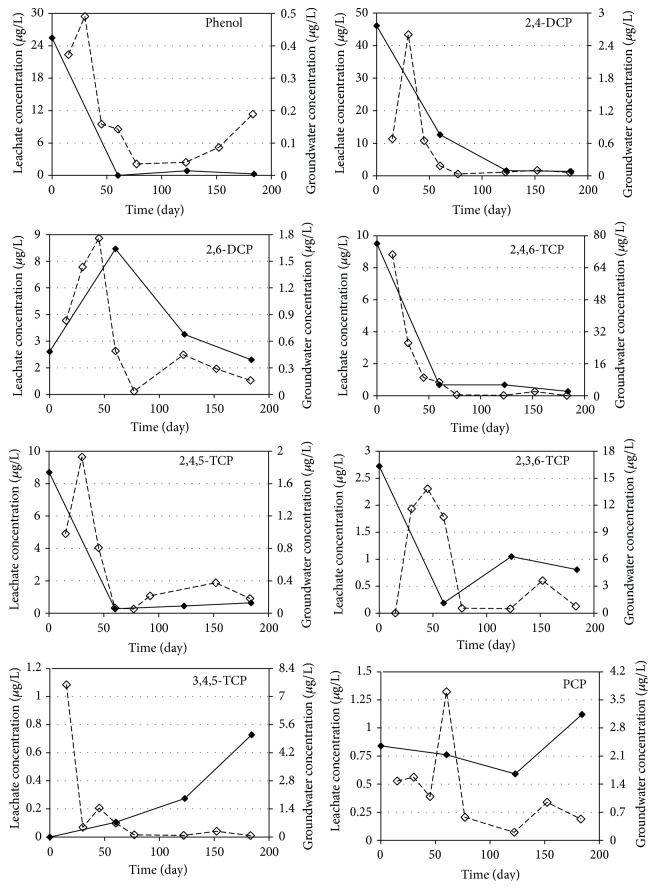
Variations of the phenolic compounds in groundwater and leachate samples of R3 reactor (——: leachate concentration, - - - - -: groundwater concentration).

**Figure 5:**
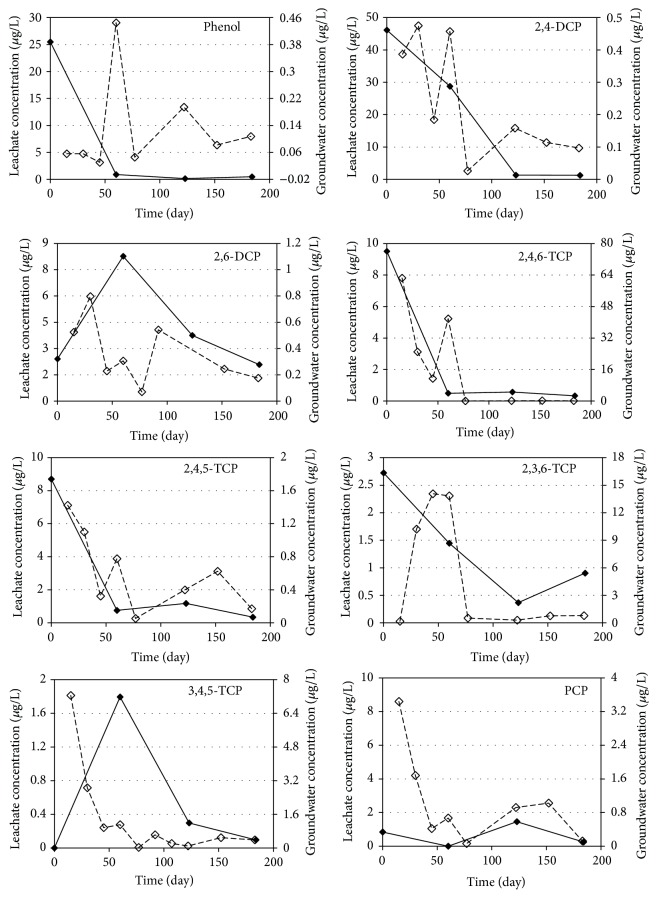
Variations of the phenolic compounds in groundwater and leachate samples of R4 reactor (——: leachate concentration, - - - - -: groundwater concentration).

The gradual changes of phenolic compounds in leachate and groundwater samples taken from R1, R2, R3, and R4 reactors are given in Figures 2 [Fig fig3] [Fig fig4]–5, respectively. The demonstrated results are the average of two measurements for each sample.

The average phenol concentrations in leachate samples of R1, R2, R3, and R4 reactors were 6.91, 6.91, 6.67, and 6.77 *μ*g/L, respectively. The observed phenol concentrations decreased in all of the reactors during the study because of the decomposition of phenolic compounds. Results indicated that phenol concentrations decreased rapidly during the first two months of operation and that phenol degradation occurs faster than that for the other phenolic compounds investigated in anaerobic medium. Phenol concentrations in groundwater samples taken from all reactors were around 0.2 *μ*g/L on average. The average migration rates of phenol from leachate to groundwater were accordingly determined to be below 2% for all reactors. Thus, it can be concluded that the change of mineral materials used in landfill liner systems will not have an effect on phenol transport to groundwater. The results also indicated that the decrease in phenol concentrations is not derived from the migration while anaerobic biodegradation and sorption may be responsible for the reduction of phenol concentrations.

The average 2,4-DCP concentrations in leachate samples during 180 days of operation were determined as 11.7, 11.6, 15.5, and 19.4 *μ*g/L for R1, R2, R3, and R4 reactors, respectively. The reduction in 2,4-DCP concentrations in leachate shows the same trend that was observed for the phenol concentrations, and the leachate concentrations decreased rapidly in the first two months of operation. The results of the groundwater analysis for 2,4-DCP resulted in average concentrations of 0.38, 0.26, 0.55, and 0.24 *μ*g/L for R1, R2, R3, and R4 reactors, indicating 3.2, 2.3, 3.6, and 1.2% of migration, respectively. The results show that zeolite (R4) and bentonite (R2) are effective materials and kaoline (R3) is the least effective liner for the prevention of 2,4-DCP transport to groundwater. It can also be concluded that sorption and biodegradation are effectively removing 2,4-DCP from leachate.

A similar trend can be seen for 2,6-DCP in leachate samples, but the decrease was not observed as fast as 2,4-DCP. The observed leachate concentrations were 3.25 *μ*g/L for R1 and R2 reactors and 3.98 and 4.12 *μ*g/L for R3 and R4 reactors, respectively. The average groundwater concentrations were also very close to each other (app. 0.4 *μ*g/L) except for the kaoline (R3) reactor which had the highest 2,6-DCP concentration (0.68 *μ*g/L). Based on the experimental results, transport percentage of 2,6-DCP from landfill leachate to groundwater was determined to be about 12.3%, 12.7%, 17.1%, and 8.7% for R1, R2, R3, and R4 reactors, respectively. Results indicated that zeolite is the most effective and kaoline is the least effective liner material for the transport of 2,6-DCP to groundwater from landfills.

Average concentrations of 2,4,6-TCP were determined as 2.92, 3.45, 2.78, and 2.73 *μ*g/L in leachate samples and 18.27, 22.83, 14.47, and 17.63 *μ*g/L in groundwater samples for R1, R2, R3, and R4 reactors, respectively. The concentrations in the groundwater samples are almost six times higher than that of the leachate concentrations [46]. The same trend was observed for the other trichlorophenol isomers except for 2,4,5-TCP. As a result of reductive dehalogenation processes, multichlorophenols are usually transformed to mono- or dichlorophenols under anaerobic conditions [47]. According to Yang et al. [48] the dechlorination rate of chlorophenols decreases with increasing number of chlorine substituents on the aromatic ring. Although anaerobic dechlorination of more highly chlorinated chlorophenols follows by anaerobic mineralization of the resulting monochlorophenols, these compounds are both prone to leaching into the water and persistent in soils [49]. Also, chlorophenols can easily migrate in groundwater because of their solubility in water [50]. It has been reported that bioaccumulation potential correlated to the octanol water partition coefficient (Kow) followed the order of 2-CP< 4-CP< 2,4,5-TCP< 2,4,6-TCP< 2,3,4,6-TeCP< PCP [49]. Therefore, it is seen that trichlorophenols (2,4,6-TCP, 2,3,6-TCP, 3,4,5-TCP, and 2,4,5-TCP) had a higher potential of migration to groundwater in all reactors. Results indicated that all of the mineral liners are not effective in preventing the trichlorophenol migration from landfills.

The average PCP concentrations for R1, R2, R3, and R4 reactors were determined as 2.26, 0.94, 0.83, and 0.64 *μ*g/L in leachate samples and 1.60, 1.58, 1.26, and 1.04 *μ*g/L in groundwater samples, respectively. PCP concentrations increased in the groundwater samples except for R1 reactor. The results indicated that PCP transport to groundwater could not be prevented by using different mineral layers from landfill liners. Previous studies have demonstrated that the removal of PCP by aerobic bacteria was not possible; however, dechlorination under anaerobic conditions would be efficient. Under anaerobic conditions, PCP is first reduced to TeCP compounds, then to TCP and DCP compounds, respectively, and finally to phenol to be mineralized [51]. In the first 100 days of operation, PCP concentrations of the groundwater samples taken from the reactors were determined to be at high levels which indicates that the mineralization process of PCP was very slow. As a general result, the concentrations of highly chlorinated phenolic compounds in groundwater samples are higher than that of the leachate samples due to the degradation and transformation of phenolic compounds. Highly chlorinated phenolic compounds demonstrated slower transformation compared to other phenolic compounds resulting in an easier migration of these compounds into the groundwater than that for the other chlorinated compounds of this study.

The dominant mechanism for the transport of phenol and phenolic compounds from leachate to groundwater is molecular diffusion. Geomembranes are ineffective in organic contaminant transport [30]. The findings indicated that only mono- and dichlorophenols migration can be prevented by mineral layers, but these layers are ineffective in delaying the transport of polychlorinated phenolic compounds. Additionally, the four mineral layers used in this study failed to prevent the transport of contaminants but that the migration of phenolic compounds to groundwater can be decreased by the use of zeolite material in landfill liners.

Adsorption can also be considered as a fundamental affecting the migration of phenolic compounds to groundwater by landfill liners. Chaouati et al. [52] showed that adsorption of phenol onto zeolites Y modified by silylation that are one of synthetic zeolites was very fast and supported at acidic conditions. Synthetic zeolites have different pore size and Si/Al ratio affecting the adsorption rates. Also they found that phenol adsorption increases with Si/Al ratio. Damjanović et al. [53] also reported that hydrophobic zeolites that possess higher contents of Si show higher affinities for phenol adsorption. In the present study, zeolite has the highest Si/Al ratio indicating the higher affinity for phenol adsorption.

## 4. Conclusion

Four lab-scale HDPE reactors with different liner materials (clay, bentonite, kaoline, and zeolite) were used in order to determine the transport of phenolic compounds in landfill leachate through liner systems. Leachate samples used in the study correspond to a middle-aged landfill leachate. The reduction of all phenolic compounds to phenol as an end product caused the high concentrations of phenol in raw leachate. According to the soil analysis, bentonite seems to have the lowest hydraulic conductivity and a higher adsorption capacity.

Experimental results indicated that the mineral materials used in landfill liners will not have an effect on mono- and dichlorophenol transport to groundwater. The observed decrease of these compounds in leachate samples was derived from biodegradation and sorption mechanisms. The reason for the high concentrations of highly chlorinated phenolic compounds in groundwater samples can be explained by the transformation of phenolic compounds under anaerobic conditions. Highly chlorinated phenolic compounds demonstrated slower degradation compared to other phenolic compounds resulting in a substantial migration of these compounds to groundwater.

As a general result, mono- and dichlorophenols migration can be prevented by mineral layers, but these layers are ineffective for polychlorinated phenolic compounds. Additionally, the four mineral layers used in this study failed to prevent the transport of contaminants. However, the migration of phenolic compounds to groundwater can be decreased with the use of zeolite material in landfill liners. It was also determined that the transport of highly chlorinated phenolic compounds from mineral layer is possible through different processes. Due to degradation and transformation activities, these compounds can be found in groundwater with higher concentrations than the leachate. Therefore, the analysis of highly chlorinated compounds such as 2,4,6-TCP, 2,3,6-TCP, 3,4,5-TCP, and PCP is of great significance for the studies to be conducted on the contamination of groundwater around landfills by leachate.

As a result of this work, the best of four different materials used may be zeolite because of high silt content and adsorption capacity. Adsorption studies by using zeolite and/or zeolite + clay mixture can be conducted to determine the best material minimizing the migration of leachate contaminants to groundwater for the future studies.

## Figures and Tables

**Table 1:** Leachate characteristics.

Parameter	Value
pH	7.8
Conductivity (mS/cm)	33
COD (mg/L)	22000
BOD (mg/L)	13000
TOC (mg/L)	6200
TKN (mg/L)	2700
TP (mg/L)	14
NH_3_ (mg/L)	2500
Cl^−^ (mg/L)	4400
SO_4_^−2^ (mg/L)	500
Phenol (*µ*g/L)	26
2,4-Dichlorophenol (*µ*g/L)	46
2,6-Dichlorophenol (*µ*g/L)	2.4
2,4,6-Trichlorophenol (*µ*g/L)	9.5
2,4,5-Trichlorophenol (*µ*g/L)	8.7
2,3,6-Trichlorophenol (*µ*g/L)	2.7
3,4,5-Trichlorophenol (*µ*g/L)	ND
Pentachlorophenol (*µ*g/L)	0.8

**Table 2:** Properties of the mineral materials used in the study.

Material	pH	Clay content (%)	Silt content (%)	Soil classification	Hydraulic conductivity (m/s)	Optimum water content (%)	CEC (meq/100 g)
Clay (R1)	7.4	85	15	CH-inorganic clays of high plasticity	6.3 × 10^−8^	27	19.3
Bentonite (R2)	8.8	89	11	CH-inorganic clays of high plasticity	2.7 × 10^−10^	41	48.6
Kaoline (R3)	7.7	57	43	ML-inorganic silts with slight plasticity	3.1 × 10^−7^	23.5	10.1
Zeolite (R4)	8.1	19	81	MH-inorganic silts with high plasticity	8.8 × 10^−8^	40	20.2
